# Generalized Cowpox Virus Infection in a Patient with HIV, Germany, 2012

**DOI:** 10.3201/eid2203.151158

**Published:** 2016-03

**Authors:** Philipp Fassbender, Sabine Zange, Sofi Ibrahim, Gudrun Zoeller, Frank Herbstreit, Hermann Meyer

**Affiliations:** Universitaet Duisburg-Essen, Essen, Germany (P. Fassbender, F. Herbstreit);; Bundeswehr Institute of Microbiology, Munich, Germany (S. Zange, S. Ibrahim, G. Zoeller, H. Meyer);; Edgewood Chemical Biological Center, Aberdeen Proving Ground, Maryland, USA (S. Ibrahim)

**Keywords:** cowpox virus, Orthopoxvirus, HIV, human, Germany, viruses

**To the Editor:** In October 2012, a 35-year-old man with clinical category C HIV infection was admitted to the intensive care unit at the University of Duisburg–Essen, Essen, Germany. The man had severe respiratory distress syndrome with septic shock, and he was infected with hepatitis B and C viruses and Epstein-Barr virus. Standard infection-control procedures were followed: the patient was placed in a single room; healthcare providers wore personal protective equipment (gown, face shield, mask, and gloves); and a closed system was used for endotracheal suctioning. 

Physical examination of the patient revealed multiple skin lesions on his right forearm and right leg. In the following days, more skin lesions appeared on his abdomen and head. The skin lesions were inflamed macules with central livid, hemorrhagic ulceration (1–2 cm in diameter) and raised edges. Kaposi sarcoma was suspected initially, but on hospital day 5, a skin biopsy showed large intracellular eosinophilic inclusion bodies pathognomonic for infection with cowpox virus (family *Poxviridae*, genus *Orthopoxviru*s). To confirm the diagnosis of cowpox virus infection, we conducted biopsies of 3 skin lesions on hospital day 7. Despite antimicrobial drug and supportive therapy, the patient died that day from septic shock. 

The 3 biopsy samples obtained on hospital day 7 were cultured on African green monkey kidney (MA104) cells, and within 2 days, many plaques were observed. DNA extracted from homogenates and virus isolated from the biopsy material were tested by orthopoxvirus real-time PCR (*1*); results were positive for all 6 samples. We confirmed the presence of cowpox virus DNA in all samples by sequencing the hemagglutinin gene.

Serum obtained from the patient on day 2 after admission, when the first lesions were noted, was also positive for orthopoxvirus DNA by real-time PCR (*1*); approximately 50 genome copies were detected, corresponding to a cycle threshold of 29.7. No orthopoxvirus-specific IgG was detected by immunofluorescence assay; this lack of detection is in agreement with observations that orthopoxvirus antibodies can first be detected in the pustular stage of disease but not as early as the macular stage (http://www.bt.cdc.gov/agent/smallpox/smallpox-biological-weapon-abstract.asp). The patient was born after the cessation of mandatory smallpox vaccination, so vaccine-induced IgG is unlikely.

Generalized cowpox virus infection in humans is atypical; the disease usually manifests as a single painful, ulcerated vesiculopustular lesion, which subsequently forms a scar, accompanied by malaise, fever, and long-lasting, painful local lymphadenopathy. However, in immunocompromised persons and persons with eczema, a generalized (and lethal, in at least 1 case) smallpox-like infection can develop (*2*–*7*). Phylogenetic analysis, based on the hemagglutinin gene, of the cowpox isolate from this study (GenBank accession no. KT182068) (Figure) revealed a close relationship with cowpox viruses isolated from humans in Germany during 1990 and 2009 (*8*). This group of viruses forms a distinct clade that is closer to taterapox, camelpox, and variola (smallpox) viruses than to another clade that contains other strains of cowpox virus and vaccinia and monkeypox viruses. Previous reports have postulated the existence of at least 2 species of cowpox virus (*8*).

**Figure:**
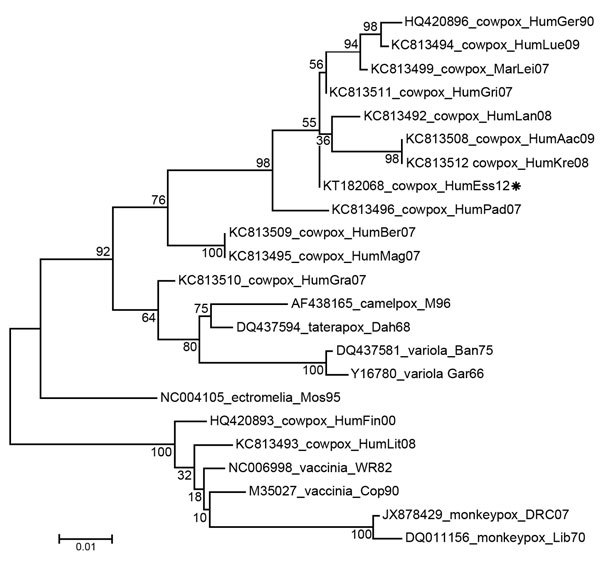
Evolutionary relationships of cowpox virus isolated from a 35-year-old man with HIV infection treated in the intensive care unit at the University of Duisburg–Essen, Essen, Germany (KT182068_HumEss12, asterisk), other human isolates of cowpox virus, and other orthopoxviruses. The evolutionary history was inferred by using the maximum-likelihood method. The percentage of trees in which the associated taxa clustered together is shown next to the branch nodes. The tree is drawn to scale, with branch lengths measured in the number of substitutions per site. The analysis involved 23 nt sequences (GenBank accession numbers are indicated). All positions containing gaps and missing data were eliminated; the final dataset had 772 positions. Evolutionary analyses were conducted in MEGA6 (http://www.megasoftware.net). Aac, Aachen; Ban, Bangladesh; Ber, Berlin; Cop, Copenhagen; Dah, Dahomey; DRC, Democratic Republic of Congo; Ess, Essen; Fin, Finland; Gar, Garcia; Ger, Germering; Gra, Graz; Gri, Grimmen; Hum, human; Kre, Krefeld; Lan, Landau; Lei, Leipzig; Lib, Liberia; Lit, Lithuania; Lue, Luedenscheid; Mag, Magdeburg; Mar, Patagonian mara (*Dolichotis patagonum*); Mos, Moscow; Pad, Paderborn; WR, Western Reserve. Scale bar indicates the number of nucleotide changes per site.

Cattle were initially incorrectly presumed to be cowpox virus reservoirs; today, wild rodents are considered to be the true reservoirs. Cowpox virus is transmitted to humans by direct contact with infected animals, mainly cats, which become infected when hunting small rodents. The incubation period is typically 7–12 days. The source of infection for the patient in our study remains unclear; interviews with his family revealed no previous contact with pet animals.

Vaccinia virus, another orthopoxvirus, is known to have induced a generalized infection in a 19-year-old military recruit after smallpox vaccination; the recruit had HIV infection, but this was not known before vaccination (*9*). Satellite ulcers at the site of inoculation and a widespread, disseminated pustular rash resulted in disseminated vaccinia and AIDS-associated complications that culminated in death of the recruit 18 months after vaccination.

In patients without underlying disease, cowpox infections manifest as self-healing diseases. However, in the absence of vaccination and among a population with increased numbers of immunocompromised persons, the risk for human poxvirus infections is increasing. Early diagnosis is essential for differentiating cowpox from illnesses and skin reactions with similar signs and symptoms, such as smallpox, monkeypox, generalized vaccinia virus infection, disseminated herpes zoster and herpes simplex virus infections, drug-associated eruptions, erythema multiforme, enterovirus infections, secondary syphilis, scabies, insect bites, impetigo, and molluscum contagiosum. The oral drug tecovirimat (previously known as ST-246), as well as cidofovir, CMX–001 (an antiviral substance), and vaccinia immune globulin, should be considered for use as postexposure therapeutic treatment for orthopoxvirus disease (*10*).
